# Voltage-Dependent Calcium Channels, Calcium Binding Proteins, and Their Interaction in the Pathological Process of Epilepsy

**DOI:** 10.3390/ijms19092735

**Published:** 2018-09-12

**Authors:** Jie-Hua Xu, Feng-Ru Tang

**Affiliations:** 1 Epilepsy Research Laboratory of Human Anatomy and Histoembryology Department, Key Laboratory of Environment and Genes Related to Diseases (Xi’an Jiaotong University), Xi’an Jiaotong University School of Basic Medical Sciences, Ministry of Education, Xi’an 710061, China; xujiehua@mail.xjtu.edu.cn; 2 Radiation Physiology Laboratory, Singapore Nuclear Research and Safety Initiative, National University of Singapore, 1 CREATE Way #04-01, CREATE Tower, Singapore 138602, Singapore

**Keywords:** calcium binding proteins (CBPs), voltage-dependent calcium channels (VDCCs), epilepsy

## Abstract

As an important second messenger, the calcium ion (Ca^2+^) plays a vital role in normal brain function and in the pathophysiological process of different neurodegenerative diseases including Alzheimer’s disease (AD), Parkinson’s disease (PD), and epilepsy. Ca^2+^ takes part in the regulation of neuronal excitability, and the imbalance of intracellular Ca^2+^ is a trigger factor for the occurrence of epilepsy. Several anti-epileptic drugs target voltage-dependent calcium channels (VDCCs). Intracellular Ca^2+^ levels are mainly controlled by VDCCs located in the plasma membrane, the calcium-binding proteins (CBPs) inside the cytoplasm, calcium channels located on the intracellular calcium store (particular the endoplasmic reticulum/sarcoplasmic reticulum), and the Ca^2+^-pumps located in the plasma membrane and intracellular calcium store. So far, while many studies have established the relationship between calcium control factors and epilepsy, the mechanism of various Ca^2+^ regulatory factors in epileptogenesis is still unknown. In this paper, we reviewed the function, distribution, and alteration of VDCCs and CBPs in the central nervous system in the pathological process of epilepsy. The interaction of VDCCs with CBPs in the pathological process of epilepsy was also summarized. We hope this review can provide some clues for better understanding the mechanism of epileptogenesis, and for the development of new anti-epileptic drugs targeting on VDCCs and CBPs.

## 1. Introduction

Neuronal intracellular calcium increase plays an important role in the triggering and propagation of seizure activity [1,2]. Ca^2+^ entry via voltage-dependent calcium channels (VDCCs) conveys the electric signals to intracellular transduction cascades in a wide variety of cells including neurons, muscle cells and endocrine cells [1,3,4]. It regulates contraction, secretion, synaptic transmission, enzyme activity, protein phosphorylation/dephosphorylation, gene transcription, and controls diverse functions including cell survival and death, as well as adaptive responses to synaptic activity [5,6,7,8,9,10,11,12,13]. Therefore, VDCCs are the key signal transducers of electrical excitability, and they convert the electrical signal of an action potential in the cell surface membrane to an intracellular Ca^2+^ transient. Alterations of VDCC functions can cause abnormality in cellular events, leading to pathological consequences. In neurons, VDCCs initiate synaptic transmission [3,14,15]. Enhanced VDCCs currents with altered properties occur in neurons of epileptic patients with Ammon’s horn sclerosis (AHS) and in the dentate gyrus granule cells of epileptic animal models [16,17,18,19]. The alteration of some VDCCs subunits in resected brain tissue of epileptic patients and samples of epileptic animal models [20] suggests the possible involvement of VDCCs in epilepsy. While some studies show severe side effects of VDCC antagonists or failure in the control of epilepsy [1,21], VDCCs are still considered as promising drug targets in the treatment of epileptic seizures, as some VDCCs antagonists are both anticonvulsive and neuroprotective [22,23,24].

Ca^2+^ dependent-signaling cascades are largely mediated by calcium-binding proteins (CBPs) [25,26], and they are essential for multiple cellular and sub-cellular processes in physiological conditions. CBPs may achieve their cellular effects through Ca^2+^-dependent or Ca^2+^-independent signaling mechanisms [27,28]. CBPs mediate Ca^2+^-dependent signaling transduction pathways and regulate Ca^2+^ influx via the VDCCs in Ca^2+^-dependent feedback mechanisms [29]. CBPs containing EF-hand Ca^2+^ binding motifs are verified to regulate high voltage-activated VDCCs [30,31,32,33,34]. Many CBPs alter Ca^2+^ kinetics directly through the regulation of VDCC properties [30,31,32,33,34]. VDCCs are co-localized with CBPs in some neurons (especially in the subpopulation of hippocampal principal cell and interneurons) [35,36], and Ca^2+^ in these neurons is controlled by the synergy of VDCCs and CBPs. With the availability of human genetic databases and advanced molecular technologies, accumulated evidence suggest that dysfunctions in CBP-mediated VDCC regulation may be one of the mechanisms leading to human diseases [29]. CBPs are also involved in buffering the intracellular calcium concentration, and they may counteract an intracellular “overload” with Ca^2+^, and protect neurons from over-excitation and neuronal damage [37].

The pathological process of epilepsy includes three periods: an acute period with initiating factors (e.g., inborn brain malformations, acquired structural brain lesions etc.), including status epilepticus, a latent period (no seizure but often with the occurrence of interictal spikes), and a chronic stage with spontaneous recurrent seizures. VDCCs and CBPs have been reported to be involved in all stages of the pathogenesis of epilepsy. Epileptogenesis is mainly related to the latent period (the transformation of healthy brain tissue into hyperexcitable neuronal networks). Interictal spikes, as the omen of seizure activity, present during this period [38,39,40]. Interictal spikes include slow and fast interictal spikes [41]. The formation of the fast interictal spikes has been demonstrated repeatedly to crucially depend on VDCC-mediated Ca^2+^ influx and may be modulated by CBPs [40,42,43,44], indicating the vital roles of both VDCCs and CBPs in the pathological process of epilepsy.

## 2. The Function and Distribution of VDCCs in the Central Nervous System

Electrophysiological studies reveal different Ca^2+^ currents designated as L-, N-, P/Q-, R-, and T-type, based on the long-lasting current and intermediate voltage dependence, which were first recorded in Purkinje neurons and cerebellar granule neurons that were resistant to subtype-specific organic and peptide Ca^2+^ channel blockers, and transient current, respectively [45,46]. The L-type VDCC family has four members, i.e., voltage-gated calcium 1.1 (Ca_v1.1_), Ca_v_1.2, Ca_v_1.3, and Ca_v_1.4. P/Q-, N-, and R-type are corresponding to Ca_v_2.1, Ca_v_2.2, and Ca_v_2.3, respectively. T-type VDCCs include Ca_v_3.1, Ca_v_3.2, and Ca_v_3.3. 

L-type VDCCs members, Ca_v_1.2 and Ca_v_1.3, shape neuronal firing and activate Ca^2+^-dependent pathways involved in the control of gene expression [47]. N-type, P/Q-type, and R-type VDCCs initiate rapid synaptic transmission, and are regulated primarily by direct interaction with G proteins and SNARE proteins, and secondarily by protein phosphorylation. T-type VDCCs, i.e., Ca_v_3.1, Ca_v_3.2, and Ca_v_3.3 are important for the repetitive firing of action potentials in rhythmically firing cells, and could be activated and inactivated more rapidly and at more negative membrane potentials than other VDCCs mentioned above [45]. T-type VDCCs abnormalities in expression and function have been linked to a range of neurological diseases, including absence seizure, epilepsy, and neuropathic pain [48].

The physiological function, and distribution of VDCCs are summarized in the Table 1 and Table 2 respectively.

## 3. VDCCs in the Pathological Process of Epilepsy

The evidence to show VDCCs in epileptogenesis has been reported four decades ago, which indicated that decreases in extracellular free Ca^2+^ concentrations might trigger seizures in the brain tissues [72,73,74]. With the small volume of the extracellular space, the decrease in extracellular Ca^2+^ may indicate at least in part, the Ca^2+^ influx through neuronal VDCCs [75]. Pieces of evidence that VDCCs may take part in the pathological process of epilepsy have been reviewed in the following tables (Table 3, Table 4 and Table 5), and their possible roles in epilepsy are summarized in Table 1.

## 4. The Function and Distribution of CBPs in the Central Nervous System

Intracellular CBPs of the EF-hand superfamily are involved in the regulation of cell function and they contribute to the control of Ca^2+^ concentration in the cytosol, and participate in numerous cellular functions by acting as Ca^2+^ transporters across cell membranes or as Ca^2+^-modulated sensors, i.e., decoding Ca^2+^ signals. The members of intracellular CBPs with EF-hand [25,26,105], such as calmodulin (CaM), parvalbumin (PV), calretinin (CR), and calbindin-D28K (CB), contain 2 to 4 functioning EF-hand Ca^2+^ binding domains [29], have important function in brain, and have been indicated to be involved in many pathological process of the central nervous system. Among the intracellular CBPs superfamily, CaM, PV, CB, and CR are particularly important due to their abundance and the specificity of their distribution in the central nervous system. These CBPs are valuable markers of neuronal subpopulations for anatomical and developmental studies, although it remains unknown whether they all play a “triggering” role like CaM, or only act as buffers to modulate cytosolic calcium transients [106]. 

Among the EF-hand CBPs, the CaM family is very large and has been extensively characterized. CaM was firstly discovered in the bovine [107] and rat [108] brain and could be found in all eukaryotic organisms. A wide range of cellular processes, including cell division and differentiation, gene transcription, DNA synthesis, membrane fusion, and muscle contraction is mediated by CaM [109]. Due to its crucial role as Ca^2+^ sensor in all types of cells, it is assumed that CaM may be involved in many pathological processes, such as epilepsy [110,111], PD, AD or rheumatoid arthritis. 

In the PV family of CBPs, PVs play an important role in cells by checking on calcium switching [112]. Because PV-expressing interneurons contribute to the maintenance and synchronization of cortical neurons activity through GABAergic synapses [113], PV has been associated with regulation of neuronal activity and PV-expressing interneurons may play a key role in numerous brain diseases, including epilepsy and complex psychiatric disorders, such as schizophrenia.

CR, as a member of the EF-hand CBPs family, was first identified in the retina. It is involved in intracellular calcium buffering, messenger targeting, cell cycle arrest, and apoptosis [114]. CR plays a crucial role in the modulation of intrinsic neuronal excitability [114] and the induction of LTP. Selective knockout of CR in mice resulted in an excess GABA release and impairment of LTP in the dentate gyrus (DG) [114,115], which was presumably due to an increased excitatory drive from CR-depleted mossy cells onto hilar interneurons [115,116].

As a high-affinity CBP, CB is expressed in the mammalian kidney, intestine, placenta, brain, peripheral nervous system, parathyroid gland, bone, and pancreas [117,118]. While the exact function of CB remains to be elucidated, current data suggest that it may play essential roles in neuronal Ca^2+^ buffer [119,120], ageing, and neurodegenerative diseases [121,122,123,124]. In the brain, CB may modulate intracellular Ca^2+^ transients and evoke Ca^2+^ signals in most neuronal groups [122,125], and therefore play a physiological role by sequestering free intracellular calcium, regulating intracellular responses to physiological stimuli and protecting neurons against Ca^2+^ mediated neurotoxicity by buffering intracellular Ca^2+^.

## 5. CBPs in the Pathological Process of Epilepsy

### 5.1. Background 

Neuronal intracellular calcium increase plays an important role for the triggering and propagation of seizure activity [1]. CBPs contribute to the control of Ca^2+^ concentration in the cytosol and participate in numerous cellular functions by acting as Ca^2+^ transporters across cell membranes or as Ca^2+^-modulated sensors, i.e., decoding Ca^2+^ signals [126]. Therefore, CBPs are very likely involved in the pathological process of epilepsy. Indeed, some members of CBPs, such as CaM, CB, CR, and PV, are implicated in the generation of seizure or epileptogenesis. 

### 5.2. CaM in the Pathological Process of Epilepsy 

**Figure 1:**
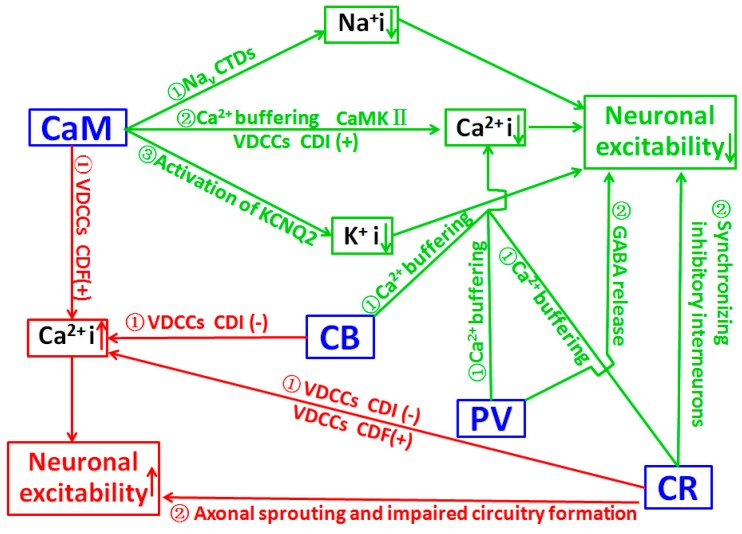
Schematic representation to show the role of calcium-binding proteins (CBPs) in the pathological process of epilepsy. The green pathways show the inhibitory role of CBPs, which may down-regulate neuronal excitability and prevent the pathological process of epilepsy; the red pathways show the excitatory role of CBPs, which likely increase neuronal excitability and promote the pathological process of epilepsy. Calmodulin (CaM) can exert inhibitory functions through three pathways, including 1. binding to intracellular C-terminal domains (CTDs) of voltage-gated Na^+^ channel (Na_v_) and limit persistent Na^+^ current and accelerate inactivation across the Na_v_ family (inhibit Na^+^ influx); 2. activation of the KCNQ2 channel (promote K^+^ efflux); 3. down-regulating intracellular Ca^2+^ through a buffering function, CaMK II media, and regulating VDCCs via Ca^2+^-dependent inactivation (CDI). calbindin (CB), parvulbumin (PV), and calretinin (CR) all can reduce intracellular Ca^2+^ through their buffering functions. In addition, PV and CR offer an inhibitory effect through regulating Gamma-amino butyric acid (GABA) release and synchronizing inhibitory interneurons respectively. CaM and CB can increase neuronal excitability by elevating intracellular Ca^2+^ via Ca^2+^-dependent facilitation (CDF) and the CDI of VDCCs respectively. CR can exert an excitatory effect through both CDF and CDI of VDCCs. In addition, CR may facilitate the pathological process of epilepsy through promoting neuronal axonal sprouting and impaired circuitry formation in the epileptic focus. (+) and (−) indicate promoting and inhibiting effects, respectively. (↑) and (↓) in the textbox indicate increase and decrease respectively. Arrows outside the textbox indicate the pathways.

The effects of Ca^2+^ are frequently mediated via its interaction with CaM, and strong evidence indicate that the effects of Ca^2+^/CaM are often achieved through the regulation of conductance of ion channels and the protein phosphorylation process [127]. A recent study suggests CaM can bind to multiple sites of voltage-gated Na^+^ channel (Na_v_) intracellular C-terminal domains (CTDs), and can limit persistent Na^+^ current and accelerate inactivation across the Na_v_ family. Mutations located in Na_v_1.2 channel CTDs can reduce CaM binding and result in increased persistent Na^+^ current and the occurrence of epilepsy [110], suggesting that CaM deficit may contribute to the pathological process of epilepsy. Epilepsy is linked to mutations in KCNQ channels, and CaM is one of the auxiliary proteins that are necessary for activation of the KCNQ2 channel [128]. CaM over-expression enhanced the outward K^+^ current and decreased neuronal activity. Meanwhile, CaM knockdown reduced the KCNQ2 current and increased neuronal activity. It suggests that CaM may regulate neuronal excitability by KCNQ2, and it could be a target of gene therapy in epilepsy [111]. These data suggest that epileptogenic factors may induce the downregulation of CaM, which in turn increases neuronal activity and results in the occurrence of epilepsy. A significant increase in CaM content in cytosol and membrane fractions of both control and kindled rats during the dark phase was related to a protective mechanism against enhanced sensitivity to seizures observed during the night, which may indicate the vital antiepileptic role of CaM [129]. CaM may also be involved in the pathological process of epilepsy indirectly through its modulation on related kinases, especially calcium/CaM-dependent protein kinases (CaMK). Transgenic mice carrying a CaMKII alpha-subunit mutation exhibit limbic epilepsy, suggesting an important CaMKII involvement in epilepsy [130]. In various seizure models, the inactivation of CaMK II occurs during seizure activity that precedes neuronal cell death [131,132,133]. Decreased mRNA level of the alpha subunit of CaMKII was found in the hippocampus of patients with intractable TLE [134]. These data may indicate that functional down-regulation of CaMKII could result in the occurrence of epilepsy. However, increased CaMKII in postsynaptic neurons was reported in KA-induced epileptic adult rat brain, [135]. In the hippocampus of patients with TLE, CaMKII labeling was significantly increased in the granule cell somata and their proximal dendrites [136]. These regional up-regulations of CaMKII may be a compensatory protective reaction to seizure activity. Collectively, the precise mechanism of CaM role in epileptogenesis through the CaMKII pathway is still unclear and needs further investigation. Both alterations of CaMKII in phosphorylation [137,138,139] and subcellular distribution [140,141] are speculated to be involved in the regulation of neuronal activity, and both alpha [142] and beta [143] subunits may be involved in the pathological process of epilepsy. The schematic presentation for roles of CaM in the pathological process of epilepsy is indicated in Figure 1.

### 5.3. CB in the Pathological Process of Epilepsy 

By a gene knockout study, the vulnerability of hippocampal CA1 neurons to seizure-induced injury was shown to be modified by CB [144]. In GEPRs, a lower level of expression in CB mRNA in the caudate putamen-accumbens nuclei was found before the induction of seizures, indicating that it might confer genetic susceptibility to, and the generalization of, seizures, in this rat strain [145]. In patients with tuberous sclerosis complex associated with refractory epilepsy, significant CB decrease was found in the cortical tubers [146]. CB immunoreactivity in the hippocampus of seizure-sensitive gerbils was significantly lower as compared with those seen in seizure resistant gerbils [147]. Loss of CB immunoreactivity in the dentate gyrus was found in patients with TLE [148,149]. Although all these data suggest the association between the decrease of CB and seizure occurrence, the causal relationship remains unknown. However, increased CB-positive cells in the epileptic brain were also reported by different research groups. Increased CB-positive neurons have been observed in the cortex of patients with focal cortical dysplasia, which might be responsible for the ongoing seizure activity [37]. In patients of TLE with microdysgenesis, many neurogliaform CB-positive nerve cells were observed in the microdysgenesis cortex [150], indicating that these CB-positive neurons might be involved in epilepsy. Furthermore, in knockout mice (PV/CB-deficient), CB did not appear to modulate the excitability of the hippocampus in a mouse model of TLE [151]. Overall, as a calcium buffer, high-level CB may bind the intracellular Ca^2+^ to reduce the overload-induced excitotoxicity and prevent the pathological process of epilepsy. The loss of CB from granule cells markedly increased the Ca^2+^-dependent inactivation (CDI) of VDCCs currents, thereby diminishing Ca^2+^ influx during repetitive neuronal firing in epilepsy [125] which may inhibit the pathological process of epilepsy. Other mechanisms, such as the synaptic reorganization of CB-positive neurons may also be involved in the pathological process of epilepsy [152]. 

### 5.4. CR in the Pathological Process of Epilepsy 

In the cerebellum, granule cells produce CR to excite Purkinje cells through parallel fibers. In vivo electrophysiological recording indicated that knocking out CR induced dramatic alterations in motor coordination and Purkinje cell firing [153]. In acute slice preparation, CR-deficient granule cells exhibited faster action potentials and generated repetitive spike discharge due to decreased calcium buffering capacity [154], suggesting that CR modulated intrinsic neuronal excitability. In the hippocampus, CR plays an important role in the modulation of neuronal excitability as CR**-/-** mice show impaired LTP induction following tetanic stimulation of hippocampal inputs, which could be restored by using bicuculline (a GABA receptor blocker) [115]. At a cellular level, except the expression of CR on mossy cells which are excitatory neurons of the dentate gyrus, CR is expressed mainly by GABAergic interneurons [155,156]. CR-positive interneurons, a distinct subpopulation of inhibitory cells innervating other interneurons in rodents, and to some extent principal cells in humans, are suggested to play a key role in the hippocampal inhibitory network, due to their function of synchronizing inhibitory interneurons [157,158,159]. The sensitivity of CR-positive interneurons to epileptic seizures has been discussed in numerous animal models, and in patients with TLE [160,161,162,163,164,165]. Increased vulnerability of CR-positive cells in patients [160,161], and in various animal models of epilepsy [166,167,168,169,170,171,172,173,174,175,176] were extensively documented, suggesting that seizure generation might be associated with a loss of a certain amount of CR-positive cells, and prevention of the loss of CR-positive cells may have antiepileptic effects. Abnormal neural circuits formed by CR-positive cells may alter the gating function of the dentate gyrus, and thereby increase hippocampal epileptogenicity [162,163,177]. The causal relationship between changes of CR-positive cells and epileptogenesis remains to be further investigated [178]. Based on current data, the role of CR in the pathological process of epilepsy has been summarized in Figure 1.

### 5.5. PV in the Pathological Process of Epilepsy 

PV is one of the CBPs that spatially and temporally controls calcium transients across the cell membranes and inside the cytoplasm. PV-positive GABAergic interneurons, which regulate the fast-spiking capability of GABAergic neurons, are responsible for mediating feed-forward inhibition within cortical networks and play an important role in the responsiveness of inhibitory neurons to an adaptation to repetitive spikes [179,180]. Therefore, changes in PV-positive interneurons may be involved in epilepsy. PV knock-out mice are more susceptible to pentylenetetrazol-induced seizures [181], suggesting the critical role of PV in the pathological process of epilepsy. A positive correlation between the presence of CB and/or PV in hippocampal neurons and their relative resistance to seizure-induced neuronal damage suggests their capability for intracellular calcium buffering [182]. Cryptogenic epileptics, who had hippocampal sclerosis as the only lesion associated with epilepsy, exhibited a preferential survival of hippocampal cells that were CB or PV positive, while other epileptics with specific aetiology lack this morphological change, indicating the possible relevance of CB and PV in epileptogenesis of cryptogenic epilepsy [183]. The Mongolian gerbil is used as a model in epilepsy studies, due to its seizure sensitivity which may be related to its specific GABAergic system, i.e., the extra (versus rat, mouse, cat and man) PV projection from the entorhinal perforant path to the hippocampus [184], which is involved in the regulation of excitatory postsynaptic potential [185]. The relative increase in the PV positive cell population in the brain may suggest the resistance of PV positive cells to cell death in certain brain regions and highlight the selective nature of neuronal loss [183,186,187]. Other studies suggest that PV-positive interneurons are extremely vulnerable to status epilepticus (SE). PV-positive cells have a homogeneous distribution in the limbic regions, including the subiculum, the entorhinal and perirhinal cortex, the hippocampus, and the dentate gyrus [187]. PV-positive interneurons are rapidly degenerated in the hilus of the dentate gyrus one day after SE [188]. A significant decrease in PV-positive neurons was found in subiculum of epileptic animals with increased subiculum network excitability [189,190]. PV-positive interneurons are also found decreased in the entorhinal cortex deep layers [191], and the perirhinal cortex [187,192] in epileptic animals with limbic network hyperexcitability. In KA-induced epileptic rat, the loss of PV interneurons may contribute to the development of spontaneous seizures as reduced PV interneuron numbers in the subiculum and entorhinal cortex is correlated with the severity of seizure occurrence [176]. The loss of PV-positive interneurons in the subiculum results in marked impairment of feed-forward inhibition of the temporo-ammonic pathway, which may significantly contribute to the occurrence of epilepsy. A decrease of PV-positive interneurons was regarded as possibly resulting from the down-regulation of PV [183,193] which might depend on the frequency by which a certain neuronal area is recruited by seizures [189]. Combining the finding that convulsive status epilepticus duration determines epileptogenesis and interictal discharge generation in the rat limbic system [194], loss functioning of the PV positive interneuron in the limbic system may play a vital role in the pathological process of epilepsy. The down-regulation of PV may lead to a profound alteration in the functional characteristics of these brain structures [189,191,192,195], such as shifting the balance of excitation and inhibition towards excitation [196], and causing an input-specific disturbance of the subicular inhibitory system [190]. Previous data also revealed a drastic loss of PV positive interneurons in the tissue from epileptic patients [187,197,198,199,200]. The loss of PV-positive interneurons in the polymorphic layer of both sclerotic and non-sclerotic epileptic patients may result in a reduced inhibition of granular cells, which in turn increases the excitability of these cells and leads to the development of uncontrolled discharges in the hippocampus. The preservation of PV positive neurons is generally attributed to the buffering capability of PV and the underlining mechanisms for a selective PV cell loss induced by SE remain to be clarified. Research indicates that PV positive cells are shown to be damaged by a mechanism independent from oxygen supply and PV positive cells in human specimens present with high mobility group box 1 (HMGB1) translocation in the cytoplasm, suggesting a mechanism involving inflammation [201]. A recent study indicated that mitochondrial fragmentation regulated by p47Phox/CDK5/DRP1 signaling pathways might be involved in the PV positive cell loss [202]. The functional impairment of the PV positive neuron was also documented [203]. In this case, the loss of AMPA receptors in surviving PV-positive neurons may impair feed-forward inhibitory outputs, and contribute to the generation of spike-wave discharges and absence seizures in stargazers. Furthermore, alterations of the specification [204], migration [205,206,207,208], maturation [172,209], excitability [210,211,212], and synaptic connectivity [213] of PV-positive interneurons in rodents with genetic disorders, and seizures and in patients with genetic epilepsy indicate the complexity of the involvement of PV in epilepsy. The generally accepted Ca^2+^ buffering and GABA release regulation are two pathways through which PV regulates neuronal excitability and epileptogenesis (Figure 1).

## 6. Regulation of CBPs on VDCCs and the Implication of the Interaction between CBPs and VDCCs in the Pathological Process of Epilepsy

CBPs such as CaM do not act as solely Ca^2+^ chelators, but they do exert an important modulatory role by regulating the delivery of Ca^2+^ signal to different substrates and contribute to Ca^2+^ homeostasis via Ca^2+^-dependent inactivation (CDI), facilitation (CDF), and Ca^2+^-independent regulation (CIR) of the channels. Pathogenesis of human diseases may occur once CBPs-mediated VDCCs are impaired [29]. The best studied CBP that regulates VDCCs is CaM. CaM contains four functional EF-hand motifs, and regulates VDCCs properties in a pattern that is similar to an enzyme inhibitor [214]. CaM binds to various high-voltage-activated VDCCs, and causes CDI [29,30,31,215,216] or CDF [29,32,34,217]. CaM-mediated Ca_v_2.1 CDF was blocked in the familial hemiplegic migraine type 1(FHM-1). This disruption of Ca_v_2.1 CDF may cause the cerebellar ataxia-associated FHM-1 due to an imbalance between the excitatory and inhibitory inputs to the cerebellar Purkinje cells [218]. Therefore, an abnormal interaction between VDCCs and CaM may be involved in the pathological process of epilepsy (Figure 1).

The interactions of CR or CB with VDCCs may also regulate neuronal excitability and be implicated in the pathological process of epilepsy. In cells expressing Ca_v_2.1 in vitro, co-expression of CR inhibits CDI and enhances CDF via a direct interaction with Ca_v_2.1. The direct modulation of Ca_v_2.1 by CR affects intracellular Ca^2+^ signaling, and probably also the neuronal excitability, via a mechanism that is different from Ca^2+^ buffering function [116]. By controlling the CDI of the L-type VDCCs, CB can regulate the amount of Ca^2+^ entry into neurons (Figure 1). Loss of CB from granule cells could markedly increase the CDI of L-type VDCC currents, thereby reducing the Ca^2+^ influx during repetitive neuronal firing in epilepsy [125]. It suggests that a low level of CB may contribute to inhibiting the pathological process of epilepsy through the regulation of VDCCs. P/Q-type VDCCs regulate neurotransmitter release in most central synapses, but they have been demonstrated to be particularly critical to sustaining GABA release from PV-positive interneurons [219]. The synaptic connectivity and properties of PV positive interneurons are partially determined by the expression of Ca_v_2.1 [220,221,222]. Many molecular determinants of PV-positive interneuron synaptic function have been associated with epilepsy [213]. Further study on the interaction between PV and P/Q type VDCC, as well as other types of VDCCs and their implication in the pathological process of epilepsy, is still needed.

## 7. Conclusions

As important pathways for Ca^2+^ entering into the cytosol and activating neuronal activity, the role of VDCCs in the pathological process of epilepsy has been well accepted. However, based on the diverse functions of VDCCs in various tissues (e.g., contraction coupling in muscles, secretion regulation in endocrine tissues, vasoactive function in the cardiovascular system, etc.) and the lack of isoform-specific inhibitors of VDCCs, clinical application of antagonists of VDCCs in patients with epilepsy has been compromised due to universal severe side effects. Recent studies have indicated that the redistribution of VDCCs may play an important role in the pathological process of epilepsy, understanding the trafficking mechanism of VDCCs becomes very important and may provide novel therapeutic targets for seizure control or prevention of epilepsy. CBPs (such as CaM, CB, PV, and CR) as Ca^2+^ buffers, can reduce Ca^2+^ overload and protect neurons from excitotoxicity, and regulate neuronal excitability through CDF and CDI of VDCCs, or other pathways. Further study to understand the molecular mechanisms of the interaction between CBPs and VDCCs in the pathological process of epilepsy will greatly facilitate the discovery of promising targets for the development of new antiepileptic drugs. By overexpression of CBPs to investigate their gain-function in reducing epileptogenesis, novel approaches in controlling or prevention of the pathological process of epilepsy may be developed. 

## Figures and Tables

**Table 1:** The distribution, physiological function, and possible roles of voltage-dependent calcium channels (VDCCs) in the pathological process of epilepsy.

VDCCs	Physiological Function	Possible Roles in Pathological Process of Epilepsy
L-Type	Shape neuronal firing and activate Ca^2+^-dependent pathways involved in the control of gene expression, and support neuronal plasticity [47]	Control neuronal excitability and likely provide the gene basis of epileptogenesis through regulation of gene expression
P/Q-Type	Regulate neurotransmitter release [49,50]	Inhibit epileptogenesis based on the fact that its null mutation can cause the occurrence of absence epilepsy
N-Type	Regulate neurotransmitter release [51,52]	Inhibit neuronal excitability through fast redistribution in the subcellullar organs of neurons
R-Type	Regulate long-term potentiation (LTP) and neurotransmitter release [53,54]	Trigger epileptiform activity in neuronal populations and promote epileptogenesis
T-Type	Regulate rhythmic firing of neurons [45]	Control burst firing of action potentials of neurons, and the plasticity of neurons induced by epiletogenic factors and promote the formation of epileptogenic focus

**Table 2:** The regional, cellular, and subcellular distribution of VDCCs in the nervous system.

VDCCs	Regional and Cellular Distribution	Subcellular Distribution
L-Type	Cav1.1 and Cav1.4 are expressed in a limited subset of neurons in the brain [55]; 90% of the L-type VDCCs in the brain are Cav1.2, and only 10% are Cav1.3 [55,56]	Located postsynaptically, predominantly in the soma, dendritic spines, and shafts of dendrites [16,19,56,57,58,59,60,61,62]
P/Q-Type	Expressed in hippocampal principal cells of the human [16] and rat [19,56,60,61,62,63], and in both hippocampal principal cells and interneurons of mice [35]	Widely expressed at the presynaptic terminals [49,50]
N-Type	Expressed in the dorsal cortex and the hippocampal formation of rats [64,65] and in both the neuron and astrocyte of the mouse brain [66]	Localized in the dendrites, presynaptic membrane, and nucleus
R-Type	Expressed in the most basal ganglia regions, the thalamus, hypothalamus, amygdala, hippocampus, and cortex [67,68,69]	Localized in the presynaptic membrane [70]
T-Type	Present in neurons in both the central and peripheral nerve system	Localized in both soma and dendrites [71]

**Table 3:** The alterations of L-type VDCCs in the animal epileptic models and patients with temporal lobe epilepsy (TLE).

L-Type VDCCs	Epileptic Animal Model	Patients with TLE
**Ca_v_1.2**	Increased in the somata of the pyramidal cells and granule cells in the KA rat model [62]; in the granule cells of the mouse pilocarpine model [35]	Increased in the astrocytes in Ammon’s horn (or hippocampal) sclerosis (AHS) specimens [16]
	Decreased in the neuropil of the CA3 stratum pyramidale and the part of CA1 regions in the KA rat model [62], in the hilar neurons of the mouse pilocarpine model [35].	Decreased in the dentate gyrus granule cells and in the residual CA3 pyramidal neurons [16]
	No changes in the hippocampal subareas in the kindling model [19]	
**Ca_v_1.3**	Increased in the hippocampal subareas in the kindling model [19], and in the granule cells of the dentate gyrus in the mouse pilocarpine model [35]	Increased in the neuropil of molecular layer of the dentate gyrus [16]
	Decreased in CA3 and the hilus of the dentate gyrus of the KA rat model [62]; in the hippocampal neurons of the kindling model [76]	

**Table 4:** The alterations of N- and R-type VDCCs in the pathological process of epilepsy and the effect of gene knockout.

VDCCs	Epileptic Animal Model	Patients with TLE	Gene Knockout Outcomes
**Ca_v_2.2**	Increased in the dendritic fields of CA1 and CA3 areas of hippocampus in the rat kindling model [77,78], in the dentate granular cells of the animal KA model [79], and in the stratum lucidum of CA3 in the mouse pilocarpine model [66].	Increased in the molecular layer [16] and granular cells of the dentate gyrus [81];	Knockout mice displayed hyperactivity and vigilance state [82]
	Decreased in the stratum lucidum of CA3 of the KA rat model [62], and in the stratum pyramidale of CA3 in the mouse pilocarpine model [66].		
	No changes in CA1 neurons in the mouse pilocarpine model [80]		
**Ca_v_2.3**	Increased in the inferior colliculus neurons of seizure-naïve rats [83];	Increased in the molecular layer of the dentate gyrus [16];	Knockout mice show hippocampal seizure resistance and reduced neuronal excitotoxicity [86,87,88]
	Decreased in both cerebellum and medulla of genetic absence epilepsy rats from Strasbourg (GAERS) [84,85];		

**Table 5:** Alterations of P/Q- and T-type VDCCs in the pathological process of epilepsy, and gene interference, mutation, and knockout outcomes.

VDCCs	Alterations in the Pathological Process of Epilepsy	Gene Interference, Mutation and Knockout Outcomes
**Ca_v_2.1**	Increased in the molecular layer of the dentate gyrus of patient with TLE [16]; different hippocampal subareas of kindling model [19]	Gene null mice exhibit ataxia and absence seizures [89]; point mutation (including tottering (tg), rocker (rkr), tottering leaner (tgla), and rolling Nagoya (tgrol)) mice exhibit reminiscent of tonic-clonic seizure, as well as electrographic and behavioral hallmarks of absence epilepsy [90]; isolated deletion in layer VI corticothalamic neurons generated absence epilepsy [91]; loss of function result in absence epilepsy [92]
	Decreased in CA3 and the hilus of dentate gyrus of the rat KA model [62]; hippocampus and neocortex of KA rat model (6 h, 24 h and 7 days after KA treatment) [61]	
	No change in the cerebellum of the rat KA model (6 h, 24 h and 7 days after KA treatment) [61]	
**Ca_v_3.1**	Increased in the reticular thalamic neurons of GAERS rats [93] and in neurons of the ventral posterior thalamic relay nuclei of adult GAERS [94]	Knockout mice did not show the burst firing of action potentials and were resistant to baclofen-induced seizures [95]; overexpression resulted in absence epilepsy [96]
**Ca_v_3.2**	Increased in both messenger RNA (mRNA) and protein level in the hippocampal CA1 area in the mouse pilocarpine model [97]	Mutation has been associated with seizure disorders, autism, and hyperaldosteronism [98]; single nucleotide mutation has been reported in patients with childhood absence epilepsy and other types of idiopathic generalized epilepsies [99,100,101,102,103,104]
